# The Love-Hate Relationship Between TGF-β Signaling and the Immune System During Development and Tumorigenesis

**DOI:** 10.3389/fimmu.2022.891268

**Published:** 2022-05-26

**Authors:** Baode Chen, Chenglin Mu, Zhiwei Zhang, Xuelin He, Xia Liu

**Affiliations:** ^1^Department of Laboratory Medicine, Key Laboratory of Clinical In Vitro Diagnostic Techniques of Zhejiang Province, The First Affiliated Hospital, College of Medicine, Zhejiang University, Hangzhou, China; ^2^Institute for Intelligent Bio/Chem Manufacturing (iBCM), Zhejiang University (ZJU)-Hangzhou Global Scientific and Technological Innovation Center, Hangzhou, China; ^3^Kidney Disease Center, The First Affiliated Hospital, College of Medicine, Zhejiang University, Hangzhou, China

**Keywords:** TGF-β, immune system, tumor progression, tumor microenvironment, cancer therapy

## Abstract

Since TGF-β was recognized as an essential secreted cytokine in embryogenesis and adult tissue homeostasis a decade ago, our knowledge of the role of TGF-β in mammalian development and disease, particularly cancer, has constantly been updated. Mounting evidence has confirmed that TGF-β is the principal regulator of the immune system, as deprivation of TGF-β signaling completely abrogates adaptive immunity. However, enhancing TGF-β signaling constrains the immune response through multiple mechanisms, including boosting Treg cell differentiation and inducing CD8^+^ T-cell apoptosis in the disease context. The love-hate relationship between TGF-β signaling and the immune system makes it challenging to develop effective monotherapies targeting TGF-β, especially for cancer treatment. Nonetheless, recent work on combination therapies of TGF-β inhibition and immunotherapy have provide insights into the development of TGF-β-targeted therapies, with favorable outcomes in patients with advanced cancer. Hence, we summarize the entanglement between TGF-β and the immune system in the developmental and tumor contexts and recent progress on hijacking crucial TGF-β signaling pathways as an emerging area of cancer therapy.

## Introduction

The immune system’s integrity and function are critical determinants of normal development and disease outcomes, particularly cancer. All immune system cells, including lymphocytic T, B, NK, and myeloid cells, originate from the hematopoietic stem cells and undergo multiple developmental stages, and TGF-β is imperative in all developmental stages. In the cancer context, the immune system and tumors evolve with unique features, making it more complicated to elucidate the multifunctional features of TGF-β during tumorigenesis. The undesirable clinical results of inhibiting the TGF-β pathway pose significant challenges for targeted therapy in cancer therapeutics. Therefore, we mainly summarize the crosstalk between TGF-β and the immune system in the context of homeostasis and disease, particularly cancer.

In both the embryonic and postnatal development stages, nearly all cells respond to TGF-β family signals and make fate decisions under the influence of TGF-β signals (1, 2). As the main components of adaptive immunity, T cells and B cells respond to TGF-β signals in different developmental stages, thus resulting in the progression or blockage of development. When mature T and B cells migrate into the periphery, they maintain peripheral immune homeostasis until they are activated by foreign antigens and differentiate into cell subsets, mostly with TGF-β involvement.

The tumor and tumor microenvironment (TME) evolve coordinately with reciprocal signaling from the tumor bulk, which consists of tumor cells, tumor epithelial cells (TECs), carcinoma-associated fibroblasts (CAFs), and immune cells. Different cells within the tumor context respond to TGF-β stimulation in a context-dependent manner and have considerable signaling heterogeneity, which is an impediment to therapeutic approaches for patients with cancer. A comprehensive understanding of the function of TGF-β in oncogenesis requires sufficient knowledge of the complicated responses of different cell types in the tumor to TGF-β. Here, we summarize current knowledge on TGF-β signaling functions in individual cellular components enclosed by the TME. Clarifying the immunosuppressive role of the TGF-β signaling pathway within tumors (3–5) and converting the tumor-suppressive microenvironment by remodeling TGF-β-initiated transmembrane signaling have spurred therapeutic progress in TGF-β-related drugs, including molecular blockers, CAR-T cells, and bispecific antibodies. We also highlight future challenges and directions in combining established regimens with anti-TGF-β to further enhance therapeutic efficacy.

## Biological Activity and Signaling Models of the TGF-β-Related Pathway

**Figure 1:**
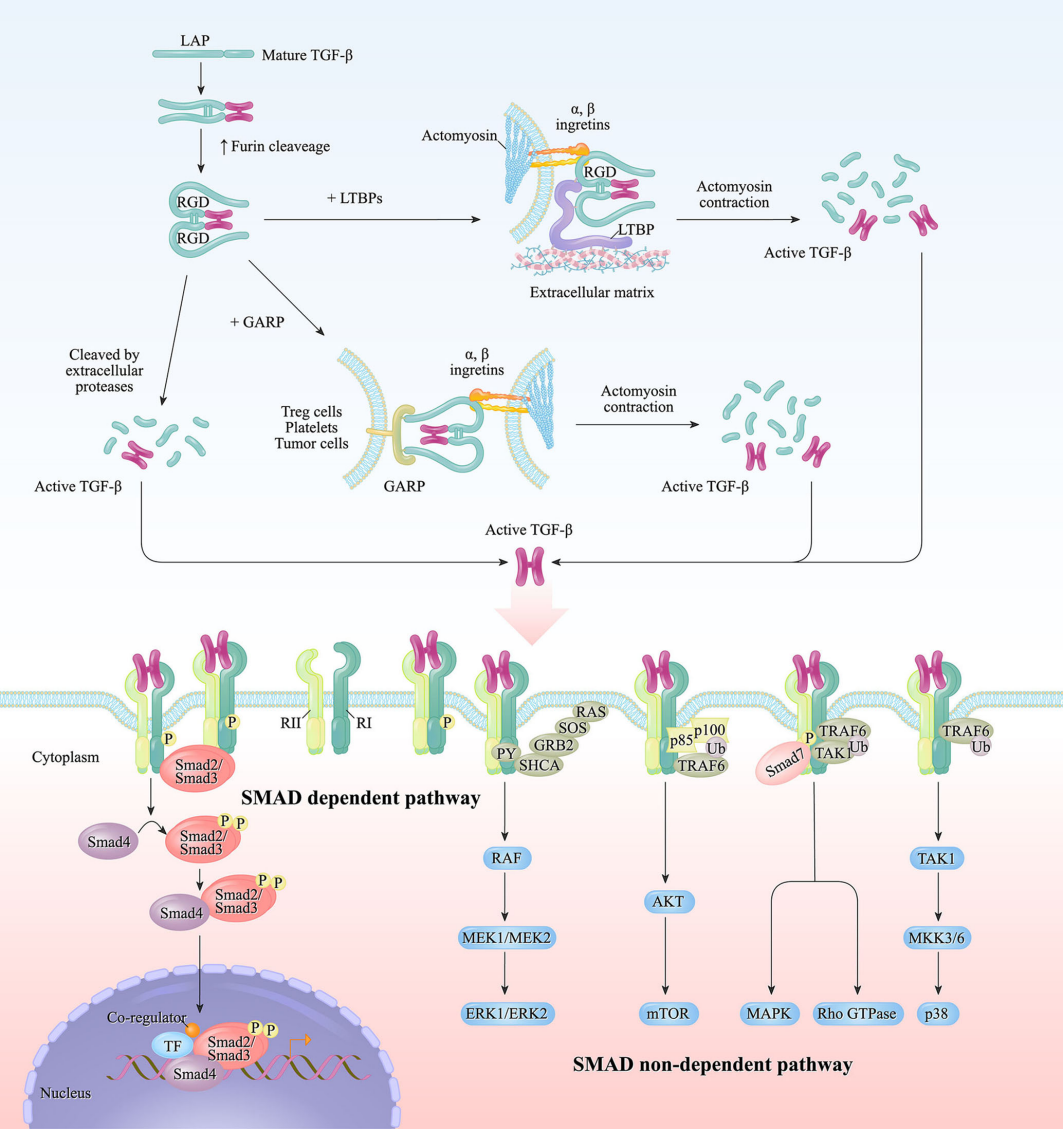
Canonical and noncanonical TGF-β signaling. Engagement between TGF-β and its receptor initiates canonical and non-canonical signaling pathways. The mature fragment of TGF-β1 remains associated with latency-associated peptide (LAP) at N-terminal propeptide. LTBPs (latent transforming growth factor β binding proteins) from the extracellular matrix (ECM) form the large latent complex (LLC) with TGF-β in the endoplasmic reticulum. When TGF-β was released and recognized by receptors, it subsequently stimulates the canonical and non-canonical pathways in cells through separate mechanisms. In canonical signaling, which is also called the SMAD-dependent pathway, the receptor activation triggers a cascade of SMAD proteins phosphorylation and translocation into the nucleus, thus promoting the downstream gene expression. While in non-canonical signaling, the receptor ligation leads to SMAD non-dependent pathway activation, including MEK/ERK pathway, AKT pathway, MAPK, and p38 pathways.

The biology of TGF-β signaling has been extensively investigated in several invaluable reviews (3, 6). **Figure 1** presents a flow diagram of TGF-β secretion and the downstream signal pathway activation process. Overall, the mammalian genome encodes three functionally overlapping TGF-β isoforms, and each isoform binds noncovalently to the latency-associated peptide (LAP) at the N-terminal portion (7, 8). LAPs associate with the large ECM protein LTBP (latent TGF-β1 binding protein) by disulfide bonds to compose a tripartite complex termed the large latent complex (LLC). The primary biological function of LAP in LLC is to confer latency to TGF-β by preventing the binding of TGF-β to TGFR1 and TGFR2, whereas LTBP mainly functions to tether bona fide latent ligand to the ECM and assist with the proper folding and secretion of TGF-β (9). The release of TGF-β from the ECM mainly occurs through extracellular proteolytic cleavage from the LAP domain dependent on proteases (7), as well as matrix metalloproteinases in the TME (10).

Released TGF-β triggers cellular signaling by engaging with the tetrameric receptor complex, which is composed of TGF-βRI and TGF-βRII. Then, TGF-βRII binds to TGF-β and recruits and phosphorylates TGF-βRI in the form of a heterotetrameric complex. Activation of the TGF-β receptor complex triggers Smad-dependent or non-Smad-mediated cascade events. In the canonical Smad-mediated model, TGF-β binds to transmembrane receptors and then recruits and phosphorylates intracellular Smad2 and Smad3 proteins, forming heterotrimeric complexes with SMAD4, which then translocates into the nucleus to activate or repress target gene transcription (11).

The SMAD-independent noncanonical TGF-β signaling pathway includes several context-dependent downstream pathways, including the ERK/MAPK, PI3K/AKT, and MKK/p38 pathways (12, 13). For example, during the epithelial-mesenchymal transition (EMT), TGF-β-modulated fibroblastic lineage reprogramming and cell emigration are dependent on p38 mitogen-activated protein kinase (14). The kinetics and functions of the ERK/MAPK pathway when encountering TGF-β stimulation in epithelial cells, fibroblasts, and cancer cells are tissue specific (15).

## The TGF-β Signaling Pathway Is Involved in the Development of the Hematopoietic and Immune Systems

**Figure 2:**
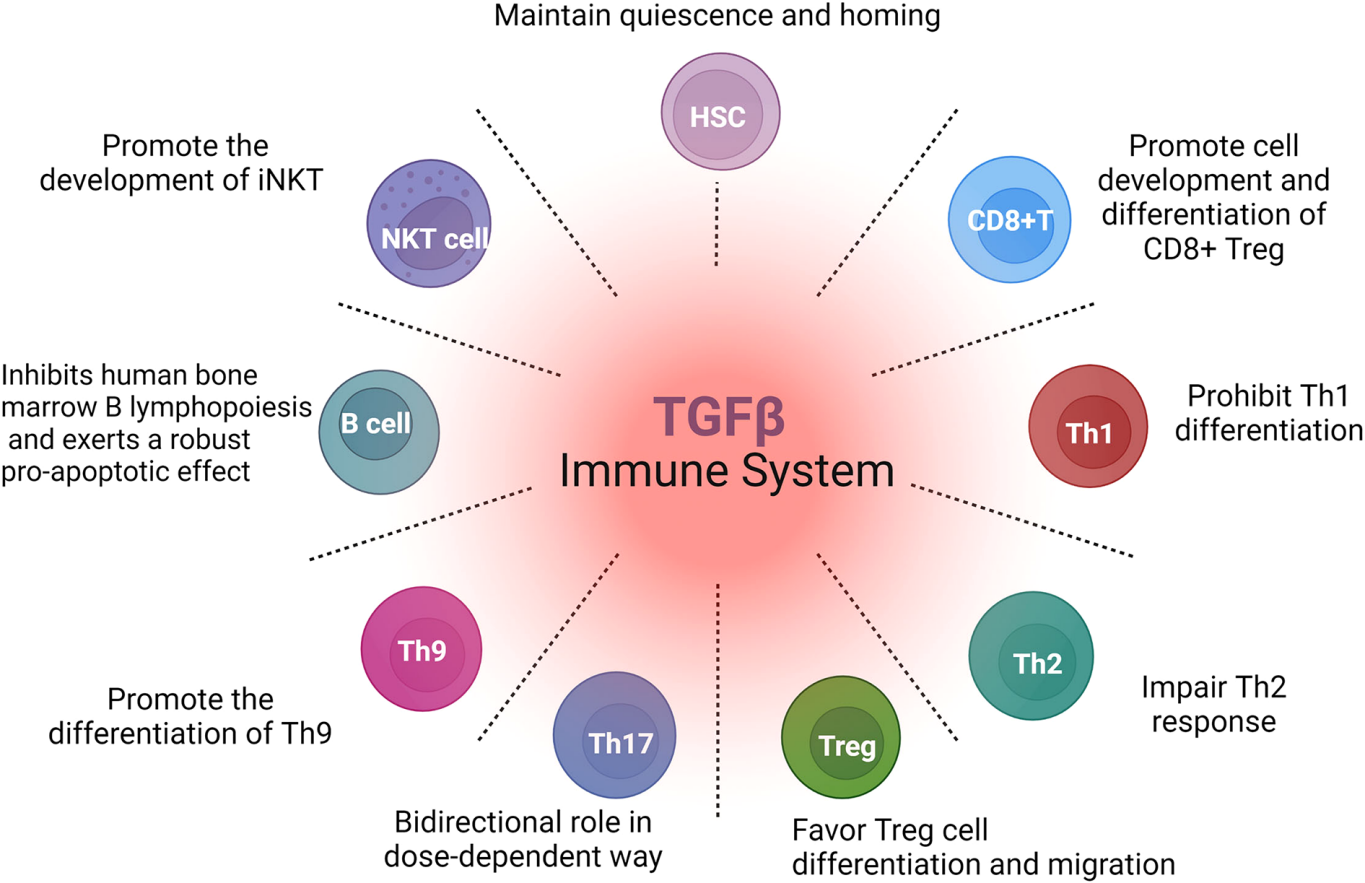
Schematic overview of the multiple effects of TGF-β on immune cells. TGF-β signaling broadly regulates the development of immune cells from the embryo to the adult. TGF-β exerts cell-specific functions in multiple immune cell components during development *via* different molecular mechanisms.

Hematopoietic stem cells (HSCs) sustain the lifelong provision of immune cells. The immune system is a complex network of biological processes that protect individuals from infection and disease. TGF-β plays essential roles in different stages of immune system development and maintains immune tolerance and cellular homeostasis by exerting specific functions on various immune cell components, as summarized in **Figure 2**.

### Hematopoietic Stem Cells

The earliest evidence supporting the role of TGF-β in mouse development was obtained by mutation of the TGF-β1 gene in embryonic stem cells, which led to mice dying by 3-4 weeks of age (16, 17). HSCs in the bone marrow were shown to preserve quiescence and infrequent division to maintain the continuous replenishment of the mature peripheral immune cell pool. Numerous subsequent studies have confirmed the role of TGF-β in the hematopoietic system in both humans and mice (18–20). In bone marrow, the ablation of megakaryocytes (MKs) significantly increases HSC proliferation through TGF-β-SMAD signaling (21). Furthermore, several groups provided evidence for the physiological influence of TGF-β in maintaining HSC quiescence, including increased HSC cycling and reduced regenerative capacity upon transplantation in TGF TGF-β RII-deficient HSCs (22), impaired HSC homing upon transplantation in TGF-β1-deficient neonates (23), and improved hematopoietic regeneration after TGF-β blockade with a neutralized TGF-β antibody (1D11) accompanied by chemotherapy (24). Interestingly, the self-renewal and differentiation capacity of HSCs do not differ between steady-state and stress conditions in mice deficient in TGF-βRI (25, 26). The higher expression level of TGF-βRII within HSCs and other TGF-βRI-independent pathways may explain the differences detected between the two mouse models. Overall, TGF-β interferes with growth mainly through the upregulation of p57^Kip2^ in the most primitive HSC compartment (27–29). In addition, TGF-β1-deficient mice display enhanced myelopoiesis, suggesting a negative regulatory role of TGF-β in myelopoiesis (30). The above observations indicate that TGF-β is a pivotal regulator of the host hematopoietic system.

### CD8^+^ T Cells

T and B cells derived from HSCs are essential components of the adaptive immune system. As the thymus is the primary site for T-cell development, early studies primarily focused on TGF-β regulation of intrathymic T-cell development. Notably, most intrathymic αβ T lymphocytes undergo a negative selection process, during which a majority of αβ T lymphocytes with high affinity for autonomous antigens are eradiated to avoid self-attack. Strikingly, TGF-β signaling-deficient DP thymocytes bypass negative selection, leading to substantial accumulation in the periphery and induction of autoimmune lesions in several organs (31). In addition, when intrathymic immature double-positive (DP) T cells differentiate into single CD8^+^ T cells, TGF-β regulates the cell surface level of IL-7Rα on CD8^+^ thymocytes and promotes CD8^+^ T cell lineage commitment by suppressing Gfi-1, a known IL-7Rα transcriptional repressor (32). A subset of CD8^+^Foxp3^+^ Treg cells that possess repressive function in the thymus are induced by TGF-β stimulation from CD8^+^Foxp3^−^ T cells (33, 34). Likewise, sustained commitment of thymus-derived CD4^+^FoxP3^+^ regulatory T cells (Treg cells) requires TGF-β signaling (35, 36). In summary, TGF-β instructs the intrathymic development of conventional CD8^+^ T cells and intrathymic differentiation of CD8^+^ Treg and CD4^+^ Treg populations. To interpret the specific role of TGF-β in peripheral mature T cells, Richard A Flavell’s laboratory generated a transgenic mouse model in which TGF-β signaling was exclusively abolished in T cells. The mice showed high infiltration of inflammatory cells in diverse organs, automatic activation of T cells, and autoimmune antibody secretion, confirming the role of TGF-β in modulating immunological balance (37). Subsequently, other laboratories constructed mice with TGF-βRII or TGF-βRI deficiency governed by the CD4 promoter, which showed severe developmental defects in T-cell lineages and autoimmune-associated lesions (35, 38). In addition, when major histocompatibility complex (MHC) class II molecules were mutated in TGF-β1-null mice, the mutation rescued the inflammatory phenotype (30), suggesting that TGF-β1 deficiency results in an uncontrolled T-cell response. Collectively, the above studies convincingly identify a dominant role for TGF-β acting directly during the T-cell development process by maintaining immunological homeostasis, affirming the significance of TGF-β in regulating T-cell activity.

### CD4^+^ T Cells

TGF-β signaling maintains cellular homeostasis in a similar way in CD4^+^ T cells by promoting the expression of IL-7Rα. However, due to the multifaceted nature of differentiation activity, TGF-β also exerts different functions on individual CD4^+^ T subsets, including Th1, Treg, Th17, and Th9 cells.

### T Helper 1 (Th1) Cells

Th1-cell differentiation is driven by the master transcription factor T-bet, which is potently inhibited by TGF-β (39). Ectopic expression of TGF-β in developing Th1 cells abolish the inhibitory effect of TGF-β (40), confirming the imperative role of TGF-β in limiting Th1-cell differentiation. Low Th1-cell activity and reduced immune cytotoxicity can forecast negative outcomes in colorectal cancer patients (41). In particular, microsatellite-instable (MSI) colon cancer patients exhibit an elevated ratio of Th1-to-naive T cells, which is inversely correlated with *tgfb* gene expression (42).

### T Helper 2 (Th2) Cells

The role of TGF-β in Th2 cells is controversial. In an early study, adding TGF-β to naive T cells led to the inhibition of Th2 cell differentiation by inducing Sox4, a transcription factor that negatively regulated the Th2 master regulator GATA-3. In addition, TGF-β was able to directly prevent Th2-associated cytokine secretion, including IL-5 secretion (43, 44). Intriguingly, when murine T cells lack both T-bet and TGF-βRII expression, they also spontaneously exhibit inflammation, which is associated with reinforced Th2 cell differentiation (45). In addition, in a TGF-β-rich context, such as the mucosa of T. muris-infected mice, the protein mina may act as an important counterbalance to the induction of Relmβ by the Th2 cytokines IL-4 and IL-13 (46). In cancer, a study that TGF-β signaling hinders Th2 cell responses that reconstruct the tumor vasculature and restrain tumor advancement (47).

### Treg Cells

Several studies have elaborated the major role of TGF-β signaling in Treg cells (36, 48). In essence, TGF-β signaling supports thymus-derived Treg cell survival by suppressing T-cell clonal deletion and promoting induced Treg (iTreg) cell differentiation in the periphery by inducing Foxp3 expression. Additionally, TGF-β directs the movement or retention of Treg cells in inflammatory tissue through several different molecular mechanisms, such as GPR15-mediated homing into the large intestine mucosa (49). Mechanistically, the engagement of TGF-β signaling promotes the binding of Smad3 to the enhancer region of Foxp3, which is called CNS1 (50, 51), to modulate Foxp3 expression. In addition, Smad2 cooperatively interacts with Smad3 in iTreg cell production (52).

### T Helper 17 (Th17) Cells

The signaling mechanisms underlying the role of TGF-β in the differentiation of the Th17-cell subset are controversial. Dan R Littman’s group proposed a dose-dependent theory in which a low concentration of TGF-β promotes Foxp3 expression, while a high concentration of TGF-β upregulates IL-23 receptor levels on CD4^+^ T cells and promotes RORγt^+^ Th17-cell commitment (53). As a TGF-β family member, BMP receptor 1α was demonstrated to suppress Th17-cell differentiation from CD4^+^ T cells because the loss of this receptor promotes the differentiation of Th17 cells and exacerbates colitis in a mouse model. By contrast, another study found that the ALK5 (TGF-β type I receptor kinase) inhibitor SB-505124 potently inhibited human Th17 differentiation *in vitro* by decreasing the gene expression of *il-17* and *rorγt* genes, along with the protein level of IL-17 (54). These pieces of evidence suggest that TGF-β family members may perform individual tasks during Th17-cell differentiation.

### T Helper 9 (Th9) Cells

T helper 9 (Th9) cells are CD4^+^ effector T cells that exert robust antitumor activities that are as strong as those of Th1 cells (55). Several studies have confirmed that TGF-β, in conjunction with IL-4, controls the differentiation of Th9 cells (56, 57). The underlying molecular mechanism includes downregulation of the expression of the DNA-binding inhibitor Id3 by TGF-β1 in conjunction with IL-4, which accelerates the elevated binding of E2A and GATA-3 to the promoter region of IL9, thus resulting in increased *Il9* gene transcription and Th9 cell differentiation (58). Th9 cell differentiation depends upon TGF-β along with IL-4, which is also explained by the molecular induction of the transcription factor PU.1 (59, 60). In addition, a recent study by Yichuan Xiao’s group reported that TGF-β collaborates with BFAR (a bifunctional apoptosis regulator) to regulate the antitumor function of Th9 cells, as BFAR-overexpressing Th9 cells display favorable antitumor efficacy. By contrast, BFAR KO significantly inhibits TGF-βR1 ubiquitination and Th9 differentiation, hence inhibiting the antitumor function of Th9 cells (61). Taken together, the above studies emphasize the importance of TGF-β in Th9 cell differentiation and function.

### B Cells

The involvement of TGF-β in B-cell development has been demonstrated extensively. In the early developmental stage, BMP-6 participates in fine-tuning of human bone marrow B lymphopoiesis by upregulating two important Smad targets, Id1 and Id3 (62). Moreover, TGF-β1 inhibits kappa acquisition in murine pre-B-cell clones (63), thereby regulating the transition between the pre-B-cell stage and the mature plasma cell stage with immunoglobulin-secreting ability. Antiproliferative effects of TGF-β in both murine and human mature B cells treated with exogenous TGF-β *in vitro* were subsequently reported (64, 65). Simultaneously, TGF-β also exerts a robust pro-apoptotic effect in human and murine B cells through Smad3-mediated Bim expression (pro-apoptotic member) (66, 67). B-cell-specific knockout mice with TGF-βRII deficiency show dramatically increased proliferation of peripheral B cells in response to a usually fragile immunogen, resulting in disrupted lifespan and conventional B cell function (68). Interestingly, mice with B-cell-specific Smad2 deficiency exhibit normal B-cell development but an impaired switch to IgA and expanded peritoneal B1a cells in Peyer’s patches (48), indicating a distinct context-dependent role of TGF-β in B-cell equilibrium. In summary, TGF-β impacts the establishment of the B-cell-based immune system from the early developmental stage to the mature functional stage.

### iNKT Cells

iNKT cells and conventional T cells arise from the same double-positive progenitor thymocytes, but unlike conventional T cells, iNKT cells mainly recognize endogenous and exogenous lipid antigens, which are presented by atypical MHC class I–like CD1d molecules. TGF-β signaling fine-tunes iNKT cell survival and development. A study in conditional knockout mice showed that TGF-β signaling regulates the precursor cell differentiation of iNKT cells, therefore impairing the maturation of iNKT cells in the thymus and in the periphery (69). A subsequent study identified the role of the TGF-β/SMAD4 axis in controlling RORγt^+^ iNKT subset development and operation during infection conditions (70). The role of the miR-17∼92 family in regulating iNKT cell development and maturation by regulating TGF-β signaling was revealed by another group (71). Overall, TGF-β drives the intrathymic growth of iNKT cells and plays an essential role in maintaining the function of mature iNKT cells in the periphery.

## Janus-Faced Regulation of TGF-β Signaling in Tumor Progression

The dual role of TGF-β in tumor progression is highly context dependent, with a tumor-suppressing role in the beginning stages of carcinogenesis and a tumor-promoting role during subsequent tumor progression. In the early stage, TGF-β signals predominantly inhibit cell proliferation, promote apoptosis (72), and maintain genome stability as a tumor suppressor (73). TGF-β expression is low in epithelial cells but increases in hyperplastic and neoplastic tissues. In noncancerous and premalignant cells, TGF-β promotes robust retardation of cell cycle progression by inhibiting late G1 phase activation by increasing the expression of CDK inhibitors, including p15^INK4^ and p21^CIP1^ (12, 27, 74, 75). Additionally, TGF-β has been postulated to stimulate apoptosis through various mechanisms; for example, in HCC tumor models, the TGF-β/SMAD axis prompts c-Myc-induced apoptosis, resulting in the abolishment of tumor initiation (76). Moreover, TGF-β triggers cell apoptosis by SMAD-modulated production of death-associated protein kinase (40) and repression of the ID family members ID1, ID2, and ID3, which determine the efficiency of cell amplification and differentiation (77, 78). In addition, oncogenic stress from RAS is involved in TGF-β triggering cell death in premalignant cells. In a murine model of aging, TβRII deficiency leads to enhanced keratinocyte motility with a decline in apoptosis (79).

Paradoxically, the TGF-β signaling pathway is highly expressed in many advanced cancers and is correlated with poor prognosis (80). TGF-β stimulates tumorigenesis *via* several mechanisms, including EMT, cell invasion, tumor metastasis, and immune suppression. EMT is a biological process in which epithelial progenitor cells undergo biochemical alteration and gradually lose polarity, resulting in enhanced migratory capacity, downregulation of cell–cell adhesion, and increased stem cell-like features (81). To induce EMT, TGF-β signaling activates the p38 and JNK pathways (5, 14) or the ERK1 and ERK2 pathways (82). Simultaneously, TGF-β signals foster cancer evolution and metastasis by stimulating tumor angiogenesis and the antitumor function of cancer-associated fibroblasts, allowing the tumor to evade antitumor immune responses in the TME (73). In bone metastases of breast cancer and prostate cancer, TGF-β elevates the expression of metastasis-related genes, including cxcr4, mmp1, and jag1 (83, 84). In mouse models of pancreatic and colon cancer, TGF-β exerts its antitumor functions primarily by delaying the transition of premalignant cells to malignant cells (12). Additionally, in a KrasG12D-mutant mouse model, the loss of SMAD4 accelerates progression to PDA by causing apoptosis in pancreatic progenitors (85). The overexpression of SMAD4 in SMAD4-deficient tumor cells inhibits tumorigenesis (13). Additionally, T-cell SMAD4 deficiency induces the automatic development of epithelial cancers in the gastrointestinal tract due to abnormally high levels of proinflammatory cytokines (86).

**Figure 3:**
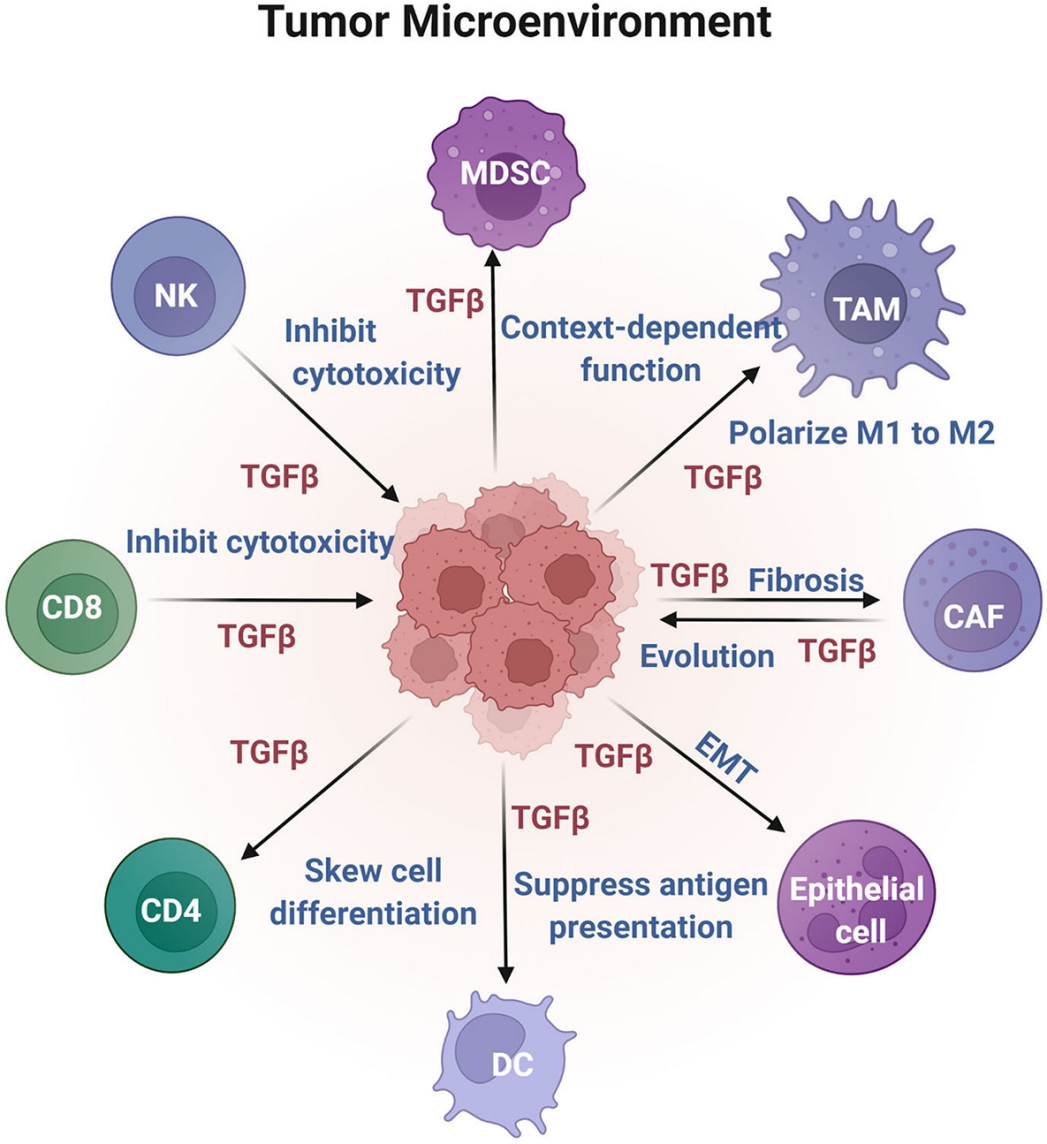
Effects of TGF-β on different cell components in the TME. TGF-β is enriched in the TME and acts on nonimmune and immune cells to fulfill antitumor or protumor growth functions.

In summary, TGF-β functions as a tumor suppressor during the initial stage of tumor formation but gradually shows its evil side as a tumor promoter with tumor progression and metastasis. In recent years, growing evidence has reinforced the immune-suppressive role of TGF-β in the TME *via* multiple mechanisms, breaking the deadlock of utilizing TGF-β inhibitors for therapeutic purposes and providing new insights and strategies for targeting TGF-β as an immunotherapy. Considering the complexity of cell components in the TME, we have summarized the function of TGF-β in individual cell subsets in the TME (shown in **Figure 3**).

## TGF-β Effects on Nonimmune Cells in the TME

In addition to cancer cells, TGF-β is strongly associated with the regulation of CAF cells, epithelial cells, and immune cells in the TME (87). The regulation of nonimmune cells is discussed in the following sections.

### CAFs

The TME is often characterized by an abundance of fibroblasts, termed CAFs, which are also the primary producers of TGF-β in various tumor types. Researchers have focused on TGF-β signaling as one of the underlying mechanisms of tissue fibrosis and tumorigenesis (88). CAFs originate from fibroblasts, which are responsive to inflammatory and tumor-derived signals (13, 89, 90). CAFs contribute to the synthesis of collagen along with the secretion of an array of soluble factors that promote tumor formation, invasiveness, metastasis (91–93), and even chemoresistance (94, 95). Elevated numbers of CAFs are regularly found in tumor patients and are negatively correlated with disease prognosis (96, 97) and immunotherapy efficacy (98, 99). In colorectal cancer patients, all colorectal cancer subtypes with poor prognosis coexpress similar gene patterns induced by TGF-β in CAFs (100, 101). A colorectal cancer tumor-bearing mouse model confirmed that increased TGF-β levels produced by CAFs in the TME represent a fundamental mechanism of immune evasion that blocks the aggregation of CD4^+^ T helper and cytotoxic CD8^+^ T cells (42). Furthermore, TGF-β blockade perturbs fibroblast activity in the TME and augments T-cell penetration and activation (102).

Mechanistically, TGF-β signaling promotes fibroblast-myofibroblast transdifferentiation through either SMAD or non-SMAD signaling pathways (103, 104). On the one hand, p53 acts as a coactivator with p-SMAD3 to induce myofibroblast production and fibrosis under TGF-β1 stimulation (105). On the other hand, the noncanonical Hippo signaling effectors YAP/TAZ cooperate with p-SMAD2/3 to drive renal fibrosis (106). In pancreatic cancers, TGF-β signaling antagonizes IL1-induced JAK/STAT activation by downregulating IL-1 receptor 1 (IL-1R1) expression and drives the commitment of iCAFs (inflammatory CAFs) to myoCAFs (myofibroblastic CAFs) (107). The crosstalk between CAFs and tumor cells through TGF-β may favor tumor progression. Although the function of TGF-β signaling in affecting CAFs has been clearly annotated, understanding the heterogeneity of the response of CAF cells to TGF-β or how to convert myCAFs into iCAFs may provide more valuable insights for unleashing the full potential of immunotherapies.

### Epithelial Cells

Resting epithelial cells rarely show TGF-β expression; however, both TGF-β1 and TGF-β receptor levels tend to be enhanced in hyperplasia and neoplasia (108, 109), indicating a tumor-promoting role of TGF-β in cancer advancement. Within tumors, elevated glucose uptake and hyperglycemia induce AKT activation and promote the externalization of TGF-β receptors, thus enhancing the cell surface presence of TGF-β receptors and TGF-β responsiveness (6). Autocrine and paracrine TGF-β represses epithelial characteristics, instructs the transdifferentiation of nontransformed epithelial cells toward a mesenchymal phenotype, and inhibits normal epithelial cell multiplication by blocking the cell division checkpoint at G1 phase (110). In response to TGF-β, epithelial cells acquire CSC-like phenotypes by concurrent hypomethylation and hypermethylation of EMT-regulating genes (8). Simultaneously, epithelial cells promote tumor progression by mitigating their epithelial characteristics and enhancing their migration and invasiveness. Moreover, TGF-β promotes epithelial plasticity by reprogramming gene expression patterns, especially enhancing EMT signature transcription factors such as Snail1, Snail2 ZEB1, and ZEB2, which cooperatively work with the SMAD3/4 complex to induce mesenchymal genes and restrain epithelial genes (111). Hence, most carcinoma cells gain proliferative properties by inactivating their epithelial antiproliferative properties and taking advantage of enhanced TGF-β signaling through effects on gene expression in epithelial plasticity.

## TGF-β Regulates the Immune Cell Response in the TME

The cellular sources of TGF-β in tumors are mainly carcinoma cells, stromal cells, and immune cells. Although most cells located in the tumor context respond to TGF-β in a context-dependent way, extensive evidence suggests that enriched TGF-β expression in the TME may compromise antitumor immunity and limit the efficacy of immunotherapy (80, 112). In the TME, TGF-β critically employs immunosuppressive functions by regulating immune populations, including adaptive B and T cells, innate natural killer cells, dendritic cells, myeloid tumor-associated macrophages, and myeloid-derived suppressor cells, as shown in **Figure 3**.

### NK Cells

TGF-β signaling within the complex tumor environment restrains the antitumor activities of assorted cell subsets, including innate immune cells and adaptive immune cells. As innate immune cells, NK cells exhibit tumor-suppressing activity by activating receptors such as NKG2D and NKp30, which are silenced by TGF-β1 directly and indirectly (113). Additionally, TGF-β silences IFN-γ and T-bet expression in NK cells (114). In TGF-β-rich TME models, TGF-β promotes the transition of NK cells into ILC1s, which are devoid of cytotoxic function (115). Moreover, SMAD4 blocks the transition of NK cells into ILC1s through noncanonical TGF-β signaling, as NK-cell-specific knockout of SMAD4 results in damaged effector NK cells and loss of metastasis control (116). Correspondingly, when tumor cells are inoculated into mice with TGF-β receptor 2 depletion in NK cells, knockout mice display significantly fewer metastases than control mice (117). In human metastatic breast cancer, TGF-β drives the metabolic malfunction of circulatory NK cells in patients (118). Blocking TGF-β and/or GARP can recover the metabolic condition and activity of NK cells, suggesting a promising strategy for enhancing NK-cell-based immunotherapies by targeting the GARP–TGF-β axis. Notably, TGF-β intensively disturbs the function of NK cells (119). The critical metabolic checkpoint kinase mTOR is one of the targets underlying TGF-β signaling in NK cells (117). In addition, pharmaceutically targeting the αv integrin/TGF-β axis in combination with allogeneic NK cells in a glioblastoma stem cell (GSC)-engrafted mouse model promotes the antitumor function of NK cells and tumor growth (120). Further studies exploring the molecular mechanisms underlying the effects of TGF-β on NK cells will provide new ideas for improving NK-cell-based therapeutics (121)

### DCs

DCs are the most powerful antigen-presenting cells and bridge the innate and adaptive immune responses by processing tumor antigens and presenting peptides to either CD4^+^ or CD8^+^ T cells. *In vitro* and *in vivo* studies have shown that TGF-β and IL-10 together suppress the maturation and activation of DCs (121). Adding TGF-β1 during the differentiation of DCs significantly amplifies the expression of DC-associated genes (122, 123) and promotes DC differentiation from progenitor CDP cells toward an mDC subset (124). In Smad7-deficient murine DC cells, the development of splenic CD8^+^CD103^+^ DCs is impaired, leading to an increased Treg cell number and resistance to autoimmune disease development (125). In mouse models of breast cancer and melanoma, increased TGF-β signaling suppresses tumor progression by increasing indoleamine 2,3-dioxygenase (IDO) in pDCs and increasing secretion of the myeloid cell attractor CCL22 (126). Moreover, blockade of both TGF-β receptor and IL-10 expression by DCs markedly enhances T-cell cytolytic activity toward cancer cells (127).

### T Cells

TGF-β impacts multiple phases of the T cell response, including activation, migration, differentiation and proliferation in both the TME and tumor-draining lymph nodes. TGF-β directly reduces CXCR3 expression on CTLs by increasing the binding of SMAD2 to the CXCR3 promoter; hence, deletion of TGF-β receptor I in CD8^+^ T cells upregulates CXCR3 expression and improves CD8^+^ T-cell trafficking into tumors (128). In addition, TGF-β reduces the cytotoxicity of CD8^+^ T cells by impairing their secretion of perforin, granzyme, and IFNγ (129), which are imperative for CTL-mediated tumor killing (130). One model proposed that TGF-β induces the phosphorylation of ITIMs, which aid the recruitment of the inhibitory protein phosphatases SHP-1 and/or SHP-2 to attenuate TCR signaling (131). This model is further supported by the multifocal lymphoproliferative inflammation phenotype exhibited by mice with TGF-β depletion in T cells (132). In addition, TGF-β impedes the secretion of IL-2, which is necessary for the proliferation and response of CD8^+^ T cells. Additionally, TGF-β1 upregulates PD-1 levels on tumor antigen-specific TILs, further providing conclusive evidence supporting the tumor-promoting role of TGF-β through the regulation of CD8^+^ T cells (133).

The role of CD4^+^ T cells in tumor eradication has drawn increasing attention in recent years. Depleting TGF-βR2 in CD4^+^ T cells halts cancer development due to tissue repair and rebuilding of the tumor vasculature (47). In a recent study, Ming O Li’s group formulated a bispecific receptor decoy named the CD4 TGF-β Trap that was able to specifically function on CD4^+^ T cells instead of CD8^+^ T cells and exert a pronounced antitumor effect (134). Among all CD4^+^ Th subsets, Th1 cells are responsible for tumor killing by cytotoxic activity. TGF-β signaling suppresses the Th1 effector regulators T-BET and STAT4 (135, 136). Furthermore, TGF-β significantly promotes the expression of the transcription factor FoxP3 and drives regulatory T (Treg) cell development (38, 45), which suppresses CTL function in the antitumor response. Moreover, TGF-β from tumor effusions mediates the inhibition of mitochondrial respiration and the generation of IFN-γ in human CD4^+^ T subsets (137). In addition, ablating TGF-βRII in CD4^+^ T cells dramatically halts cancer progression (47). Most importantly, TGF-β skews the differentiation direction of TH1 cells into TH2 and TH17 cells (138).

### TAMs

Myeloid cells are the main component of tumor-infiltrating leukocytes involved in tumorigenesis and are termed tumor-associated macrophages (TAMs). Importantly, TGF-β also affects the myeloid cell repertoire within tumors, including macrophages, MDSCs, and neutrophils. Accumulating evidence strongly indicates that the TME polarizes macrophages from the M1 to the M2 immunosuppressive protumoral subset, leading to immunosuppression and tumorigenesis. Additionally, TAMs can promote tumor cell migration and metastasis *via* the TGF-β2/NF-κB/Kindlin-2 pathway (139, 140). Therefore, TAMs are potential targets for tumor therapy (141). Malignant cells in the tumors generate TGF-β, thus upregulating CXCR4 on the monocyte cell surface, whereas perivascular fibroblasts produce the CXCR4 ligand CXCL12 to target these monocytes to the tumor, followed by monocytes differentiating into perivascular macrophages (142). Additionally, TGF-β signaling represses the anti-inflammatory role of macrophages through degradation of MYD88 to inactivate NF-κB signaling (143). Moreover, genetic mice with myeloid cell-specific depletion of Tgfbr2 display less tumor metastasis (144).

### MDSCs

Patients or mice bearing tumors always exhibit an increased number of circulating neutrophils, which are associated with a poor prognosis (145). The phenotypes of MDSCs (myeloid-derived suppressive cells) are similar to those of monocytes and immature neutrophils in chronic infection, inflammation, or cancer. The functional difference between MDSCs and neutrophils is that T-cell proliferation suppression is mediated by MDSCs and not neutrophils (146). Therefore, it is broadly accepted that MDSCs significantly repress the cytotoxic function of effector cells and promote tumor angiogenesis and metastasis (147). The function of TGF-β on MDSCs in tumor evolution has been reported by several groups. In one study, TGF-β fine-tuned MDSC accumulation and activation in tumors by inducing microRNA-494 expression (147). Additionally, TGF-β directly affects monocytic-MDSC (Mo-MDSC) expansion and MDSC functions (148). This latter finding was further supported by results in melanoma and breast cancer mouse models, which showed that TGF-β affects the generation of the CXCR3 ligand CCL9 in MDSCs, leading to less immune cell accumulation and cancer cell survival (149). In addition, conditional knockout mice with Tgfbr2 deletion in myeloid cells are resistant to tumor metastasis as a result of MDSC dysfunction, implying a critical role of TGF-β in MDSCs during tumor metastasis (144). However, restricting TGF-β signaling in the tumor epithelium induces prominent accumulation of CCR1^+^ immature myeloid cells, which increase extension and penetration by cancer cells (150). Similarly, abrogating Tgfbr2 expression in carcinoma cells results in direct recruitment of migrating MDSCs into tumors *via* the upregulation of the SDF-1/CXCR4 and CXCL5/CXCR2 axes (151). The context-dependent function of TGF-β signaling in tumor-infiltrating MDSCs remains controversial, and the molecular mechanism needs to be explored.

### Preclinical Studies Targeting TGF-β as a Cancer Therapy

The TGF-β/SMAD signaling pathway has been demonstrated to be an essential immune envision mechanism in both hematopoietic and solid tumors (152). In childhood B-ALL patients, TGF-β1 induced NK cell dysfunction to mediate escape from immune surveillance (153). In addition, TGF-β signaling blockade inhibited the proliferation of leukemia stem cells (154). In a mouse model with cancer-related anemia, pharmaceutically blockade of TGF-β signaling mitigated disease progression, suggesting a likely therapeutic target for alleviating hematopoiesis disease. Superfluous TGF-β in the bone marrow microenvironment impaired the bone marrow niches, which maintain the stemness and function of hematopoietic stem cells (155). CAR-T cell therapy has essentially reformed the therapeutic regimen of hematological malignancies (156). By constructing novel CD19 CAR-tTRII-I7R-T cells, which convert the TGF-β signaling into immune-activating IL-7 signaling, the tumor-killing efficacy of modified CAR-T cells was significantly better than in the control group (157). The above studies proposed that TGF-β signaling prohibition could be a potential therapeutic approach for relieving defects in hematopoiesis.

Solid tumors show substantial antigen heterogeneity (156). Moreover, solid tumor cells are organized into multiple compartments and are often surrounded by other tissues, where they are less accessible to T cells. Tumor-bearing mouse models show that combined treatment with TGF-β inhibition and immune checkpoint antibodies such as anti-PD-L1 were able to induce better tumor regression and more prolonged survival (42, 99), leading the way to combining TGF-β inhibitors with ICB drugs clinically for advanced cancer patients. Besides, as a newly developed fusion protein targeting both TGF-β and PD-L1, Bintrafusp alfa (M7824) was demonstrated to effectively reduce the breast tumor and colon tumor growth in mice model (158). Hence, the clinical efficacy of M7824 in different advanced malignancies was evaluated in several ongoing clinical trials. Moreover, bifunctional antibody–ligand traps (Y-traps), which target both CTLA-4 and, resulted in significantly superior antitumor efficacy compared to CTLA-4 antibody monotherapy (159). When combining specific TGF-β1 inhibitor SRK181-mIgG1 with anti–PD-1 antibody in multiple mouse models of cancer, the combo therapy significantly induced the intratumoral infiltration of CD8^+^ T cells with less dose-limiting toxicology (160). These preclinical results provide a principle for exploring TGFβ inhibition to work synergistically with ICB in cancer patients.

### Current Clinical Trials Targeting TGF-Beta in the Tumor Microenvironment

With a growing number of preclinical studies demonstrating the role of TGF-β in suppressing immune response in different tumor types, more clinical trials have been conducted recently to evaluate the therapeutic safety and efficacy of targeting TGF-β in advanced cancer patients (73, 161). Up to now, however, both promising and undesirable outcomes have been revealed. We have summarized the finished clinical trials and ongoing trials that target TGF-β signals in advanced tumor patients in recent ten years (as shown in **Tables 1**, **2**). Among them, Galunisertib (LY2157299) is a selective molecule inhibitor of RI kinase, which reduces signaling in the -rich immunosuppressive tumor microenvironment (162). A Phase I Study of Galunisertib in advanced tumor patients evaluated the safety and recommended the dose for the phase II study (163). Nevertheless, the co-administration of Galunisertib and anti-PD-L1 antibody in recurrent/refractory metastatic pancreatic cancer showed no apparent clinical activity (164). Another selective small-molecule inhibitor Vactosertib (TEW-7197), which serves as a TGF-β R1 inhibitor, was well-assessed in its pharmacokinetics (165). In a Phase Ib trial for relapsed multiple myeloma, the combo therapy with vactosertib and pomalidomide showed better efficacy assessment than historical control, indicating the further application of Vactosertib in clinical trial multiple myeloma (166). In addition, several multi-center phase 2 studies for subjects with advanced or metastatic tumors are ongoing to estimate the safety and efficacy of vactosertib in association with pembrolizumab (NCT04515979) in lung cancer patients or vactosertib plus imatinib (NCT03802084) in desmoid tumor. Moreover, a pan-anti- neutralizing antibody, NIS793, has overcome the resistance of checkpoint blockade immunotherapy in the treatment of squamous cell carcinomas (167), which paves the way for the clinical trial of NIS793/α-PD-1 combination therapy (NCT02947165). Meanwhile, this antibody is presently being evaluated together with gemcitabine/nab-paclitaxel chemotherapy and anti-PD-1 antibody for patients with metastatic pancreatic ductal adenocarcinoma in a phase II clinical trial (NCT04390763). Another pan- antibody SAR439459 is being estimated for its safety and antitumor activity either as monotherapy or together with the anti-PD-1antibody in patients with advanced solid tumors (NCT03192345).

**Table 1:** Completed clinical trials to evaluate TGF-β pathway antagonists.

Target	Agent	Tumor type	Clinical efficacy	Starting date	Identifier
TGF-β2	AP12009 Temozolomide PCV	Glioblastoma Anaplastic Astrocytoma	Not reported	2007-02-06	NCT00431561
TGF-β2	Lucanix	Non-small cell lung cancer	Mos:20 versus 17 m	2008-05-13	NCT00676507
TGF-β2	AP 12009	Pancreatic Neoplasms Melanoma Colorectal Neoplasms	Not reported	2009-02-13	NCT00844064
CT RT TβRI	LY2157299 Radiation Temozolomide	Malignant Glioma	mOS:18.2 versus 17.9 m	2010-10-13	NCT01220271
TβRI	LY2157299 Sorafenib Ramucirumab	Hepatocellular Carcinoma	mPFS 2.7 m part A and 4.2 m part B	2010-11-24	NCT01246986
TβRI	Galunisertib Gemcitabine Placebo	Advanced or Metastatic Unresectable Pancreatic Cancer	mOS 8.9 versus 7.1 m	2011-06-14	NCT01373164
TGF-β1 TGF-β2 TGF-β3	Fresolimumab RT	Refractory breast cancer	ORR 0%	2011-07-25	NCT01401062
TβRI	LY2157299 monohydrate Lomustine Placebo	Glioblastoma		2012-04-20	NCT01582269
TβRII	LY3022859	Advance solid tumors	Not reported	2012-07-20	NCT01646203
TβRI	LY2157299 Gemcitabine	Inoperable or metastatic pancreatic cancer	ORR 0%	2014-06-03	NCT02154646
TβRI	TEW-7197	Advanced Stage Solid Tumors		2014-06-10	NCT02160106
TβRI	LY2157299 Sorafenib	Metastatic hepatocellular carcinoma	ORR 9%	2014-09-15	NCT02240433
TGF-β RI	LY2157299 Lomustine	Glioma	ORR 14%	2012-09-10	NCT01682187
TGF-β RI	Galunisertib Durvalumab	Metastatic Pancreatic Cancer	ORR 3%	2016-04-12	NCT02734160
TGF-β RII PD-L1	M7824	Human Papilloma VirusCervical Cancer Oropharyngeal CancerAnal Cancer Vaginal or Penile Cancer	ORR 39%	2018-02-09	NCT03427411
TGF-β RII PD-L1	M7824	Advanced Adenocarcinoma of the Pancreas	Study was closed after one treatment related death.	2018-03-02	NCT03451773
TGF-β RII PD-L1	MSB0011359C	Metastatic or Locally Advanced Solid Tumors		2015-08-07	NCT02517398
TGF-β RII/PD-L1	M7824	Pre-treated cervical tumors	ORR 28%	2015-08-07	NCT02517398
TGF-β RII/PD-L1	M7824	Refractory head and neck cancer	ORR 22%	2015-08-07	NCT02517398
TGF-β RII/PD-L1	M7824	Pre-treated NSCLC	PD-L1 > 1%, ORR 40%, PD-L1 > 80%,ORR 71%	2015-08-07	NCT02517398
TGF-β RII PD-L1	M7824	Pre-treated esophageal adenocarcinoma	ORR 20%	2015-08-07	NCT02517398
TGF-β RII PD-L1	M7824	Pre-treated gastric cancer	ORR 22%	2015-08-07	NCT02517398
TGF-β RII PD-L1	M7824	Pre-treated biliary tract cancer	ORR 23%	2015-08-07	NCT02517398
TGF-β RII PD-L1	M7824	Refractory colorectal cancer	ORR 3.4%	2015-08-07	NCT02517398
Vaccine Anti-PD-1	Vigil Pembrolizumab	Melanoma		2015-10-14	NCT02574533

The underlined NCT Number represents the National Clinical Trial number, which is an identification that ClinicalTrials.gov assigns a study when it is registered. The NCT number is assigned when the study is registered.

**Table 2:** Ongoing clinical trials to evaluate TGF-β pathway antagonists.

Target	Agent	Tumor type	Starting date	identifier
TβRI AR	Galunisertib Enzalutamide	Metastatic Castration-resistant Prostate Cancer	2015-05-22	NCT02452008
α-TGF-β α- PD - 1	NIS793 PDR001	Breast Cancer Lung Cancer Hepatocellular Cancer Colorectal Cancer Pancreatic Cancer Renal Cancer	2016-10-27	NCT02947165
TGF-βR1 ALK5	Vactosertib Pomalidomide	Multiple Myeloma	2017-05-08	NCT03143985
TGF-β PD-L1	M7824	Pretreated MSI-H mCRC	2018-02-19	NCT03436563
PD-L1 TGF-β	M7825	Breast Cancer	2018-05-14	NCT03524170
PDL1 TGF-β	M7824 Eribulin Mesylate	TNBC	2018-07-06	NCT03579472
PD-L1 TGF-β	M7824 Topotecan Temozolomide	SCLC	2018-06-13	NCT03554473
TGF-β	Vactosertib imatinib	Desmoid Tumor	2019-01-14	NCT03802084
TGF-β PD-L1	Platinum-based regimen + M7824	Metastatic NSCLC	2019-02-15	NCT03840915
TGF-β PD-L1	M7824 With cCRT	NSCLC	2019-02-15	NCT03840902
TGF-βR1	Pembrolizumab vactosertib	Colorectal Cancer Resectable Hepatic Metastases	2019-02-18	NCT03844750
TGF-βR1	Vactosertib	Myeloproliferative Neoplasm	2019-09-25	NCT04103645
PD-L1/TGF-β	M7824	Local-Regionally Recurrent Head and Neck Squamous Cell Carcinoma	2020-01-07	NCT04220775
PD-L1 TGF-β	M7824	Cervical Cancer	2020-01-29	NCT04246489
TGF-β PD-1	Gemcitabine nab-paclitaxel NIS793 Spartalizumab	First-line Metastatic Pancreatic Ductal Adenocarcinoma	2020-05-18	NCT04390763
PDL1 TGF-β	M7824	Thymoma Thymic Carcinoma	2020-06-05	NCT04417660
PD-L1 TGF-β	M7824	Thymic Epithelial Tumor	2020-06-05	NCT04417660
PD-L1 TGF-β	M7824	Recurrent Thymoma	2020-06-05	NCT04417660
PD-L1 TGF-β	M7824	Thymic Cancer	2020-06-05	NCT04417660
PD-L1 TGF-β	PRGN-2009 M7824	Cervical cancers p16+ Oropharyngeal cancers Anal cancers Other locally advanced or metastatic solid tumors (e.g. lung, esophagus) that are known HPV+.	2020-06-16	NCT04432597
TGF-βR1 ALK5	Vactosertib 300 mg BID pembrolizumab 200 mg IV	Non-Small-Cell Lung	2020-08-17	NCT04515979
PD-L1 TGF-β	M7824 NHS-IL12 Entinostat	Cervical Oropharyngeal Anal Vulvar Vaginal	2021-01-14	NCT04708470

The underlined NCT Number represents the National Clinical Trial number, which is an identification that ClinicalTrials.gov assigns a study when it is registered. The NCT number is assigned when the study is registered.

Although significant progress was achieved, suspension or failure of anti- related clinical studies occurs in certain conditions. LY3022859 is a human anti-RII IgG1 monoclonal antibody, which significantly inhibited cancer cell growth and metastasis in preclinical models (168). However, in a phase I study of advanced tumor patients, owing to the burst of cytokine release syndrome, the study was stopped without determining the maximum tolerated dose (169). Moreover, a first-in-class bifunctional fusion protein Bintrafusp alfa (M7824), which targets both TGF-β and PD-L1, showed disease control regardless of PD-L1 in recurrent glioblastoma patients (170). Also, in biliary tract cancer patients, the M7824 monotherapy showed encouraging efficacy with durable responses (171). However, in the following phase III lung cancer clinical trials, treatment with Bintrafusp alfa was unable to show compelling efficacy, accompanied by the termination of three related trials till now. Even so, more than ten clinical trials related to Bintrafusp alfa combination therapy are ongoing in different cancer patients, indicating cancer context-dependent efficacy may be observed.

Immune checkpoint blockade therapies have achieved massive success in treating a variety of cancers. However, checkpoint inhibitors work to rejuvenate the body’s immune activity instead of removing the immunosuppressive barriers in the tumor microenvironment (159). Subsequently, the efficacy of ICB therapies is limited in a minority of patients. Hence, the TGF-β pathway inactivation has emerged as a working partner for cancer patients with ICB resistance (160).

## Discussion

The determining function of TGF-β in maintaining immune system integrity is to preserve immune homeostasis and tolerance by regulating immune cell development, proliferation, differentiation, and survival. A competent immune system must maintain an assorted pool of naïve immune cells with the companion of various cytokines, including TGF-β. Malfunctions of TGF-β block immune cell development and function, resulting in cancer progression.

Because of the pleiotropic effects of TGF-β on both normal physiological function and tumorigenesis, long-term blockade of TGF-β and the related signaling pathways may have adverse effects. Furthermore, the biology of solid tumors is complex. Therefore, the current significant challenge in translating anti-TGF-β inhibition into clinical treatment is to explore the various function of the TGF-β signal pathway acting on different cell components in the tumors, thus searching for a precise targeting approach with less toxicity and other side effects.

Importantly, TGF-β serves as a primary immune evasion mechanism in various malignancies by building a tolerogenic immune environment. One hallmark of TGF-β’s evasion mechanism is promoting the amplification and aggregation of Treg cells in the tumors, which inhibit the cytotoxicity of CD8^+^T and Th1 cells. Besides, the TGF-β can be secreted and functions on stromal cells by an autocrine pathway and promote myelofibrosis and angiogenesis. Lately, the metabolic regulating role of TGF-β in inducing CAF and endothelial cell generation in cancer was revealed, indicating a novel role of TGF-β signaling in reprogramming the metabolic landscape of the tumor environment.

Cancer immunotherapy has indeed benefited patients who cannot receive surgical therapy or are resistant to chemotherapy. Repressing TGF-β signaling has shown a synergistic effect with immune checkpoint inhibitors in preclinical models and certain patients, providing a new solution for patients who are insensitive to ICB treatment. Moreover, modified CAR-T cell therapy by depleting the TGF-β signal in CAR-T cells also demonstrates potent efficacy in treating cancers, indicating a promising field by generating more robust and less toxic CAR-T cells by modifying the TGF-β signal pathway.

Given the essential role of the TGF-β signal pathway in physiological and pathological conditions, it’s expected that the integral blockade of the TGF-β signal pathway resulted in adverse effects that restrict the therapeutic progression. Hence, new therapeutic modalities with more precise targeting or more assorted regimen design still need further exploration. In the future, specific targeting TGF-β in certain types of immunosuppressive cell components may reduce the incidence and hardness of adverse effects and increase beneficial efficacy.

TGF-β signal pathway was hyperactivated in colon cancer and pancreatic cancer. However, different tumors harbor diverse tumor microenvironments, either with low levels or high levels of TGF-β. Accordingly, more studies are needed to explore whether the tumor architecture or landscape influences the efficacy of TGF-β inhibition. Besides, whether genetic, epigenetic, and microbiota differences in different cancer types define the efficacy of TGF-β signal pathway blockade also needs to be studied. All things considered, we will be able to reach a more precise and personalized modality for treating cancers by anti-TGF-β pathway.

## Author Contributions

BC, CM, and ZZ wrote the manuscript. XH and XL wrote and critically revised the manuscript. All authors contributed to the article and approved the submitted version.

## Conflict of Interest

The authors declare that the research was conducted in the absence of any commercial or financial relationships that could be construed as a potential conflict of interest.

## Publisher’s Note

All claims expressed in this article are solely those of the authors and do not necessarily represent those of their affiliated organizations, or those of the publisher, the editors and the reviewers. Any product that may be evaluated in this article, or claim that may be made by its manufacturer, is not guaranteed or endorsed by the publisher.
